# Formative research to develop a lifestyle application (app) for African American breast cancer survivors

**DOI:** 10.21633/jgpha.6.103

**Published:** 2016

**Authors:** Selina A. Smith, Mary S. Whitehead, Joyce Q. Sheats, Brittney Fontenot, Ernest Alema-Mensah, Benjamin Ansa

**Affiliations:** 1 Institute of Public and Preventive Health, Augusta University, Augusta, GA; 2 Department of Family Medicine, Medical College of Georgia, Augusta University, Augusta, GA; 3 SISTAAH Talk Breast Cancer Support Group, Mami, FL; 4 Department of Community Health and Preventive Medicine, Morehouse School of Medicine, Atlanta, GA

**Keywords:** Lifestyle modification, intervention mapping, cancer prevention guidelines, breast cancer survivors, smartphone app

## Abstract

**Background:**

There is a proliferation of lifestyle-oriented mobile technologies; however, few have targeted users. Through intervention mapping, investigators and community partners completed Steps 1–3 (needs assessment, formulation of change objectives, and selection of theory-based methods) of a process to develop a mobile cancer prevention application (app) for cancer prevention. The aim of this qualitative study was to complete Step 4 (intervention development) by eliciting input from African American (AA) breast cancer survivors (BCSs) to guide app development.

**Methods:**

Four focus group discussions (n=60) and three individual semi-structured interviews (n=36) were conducted with AA BCSs (40–72 years of age) to assess barriers and strategies for lifestyle change. All focus groups and interviews were recorded and transcribed verbatim. Data were analyzed with NVivo qualitative data analysis software version 10, allowing categories, themes, and patterns to emerge.

**Results:**

Three categories and related themes emerged from the analysis: 1) perceptions about modifiable risk factors; 2) strategies related to adherence to cancer prevention guidelines; and 3) app components to address barriers to adherence. Participant perceptions, strategies, and recommended components guided development of the app.

**Conclusions:**

For development of a mobile cancer prevention app, these findings will assist investigators in targeting features that are usable, acceptable, and accessible for AA BCSs.

## INTRODUCTION

Among African American (AA) women in the U.S., breast cancer is the most commonly diagnosed cancer, and the second most common cause of cancer death. In 2016, an estimated 30,700 new cases and 6,310 deaths from breast cancer are expected to occur among AA women (American Cancer Society (ACS), 2016). Incidence rates continue to increase in AA women at about 0.5% per year, and death rates are 42% higher compared to White women (ACS, 2016). The increase in incidence and mortality rates in AA women may in part reflect the rising prevalence of obesity in this group. AA women have the highest rates of being overweight or obese compared to other ethnic groups in the U.S. Recent national data show that 82.0% of Black women are overweight or obese compared to 63.2% of White women (National Center for Health Statistics ((NCHS), 2016).

Obesity is a risk factor for breast cancer recurrence and poor survival (Strickler, 2013). Healthy lifestyles (e.g., keeping physically active and healthy diet) are widely recognized as contributors to cancer survivorship. Health organizations such as the American Cancer Society (ACS) and the World Cancer Research Fund/American Institute for Cancer Research (WCRF/AICR) routinely publish dietary and lifestyle recommendations aimed at cancer prevention (Kushi et al., 2012; WCRF/AICR, 2007). Both the ACS guidelines and the WCRF/AICR recommendations are concerned with maintaining a healthy weight throughout life, consuming a plant-based diet, adopting a physically active lifestyle, and limiting consumption of red meat and alcohol. Adherence to the ACS dietary guidelines and the WCRF/AICR guidelines is associated with a decrease in the incidence of cancer and mortality (McCullough et al., 2011; Hastert et al., 2013).

Current approaches aimed at modifying lifestyle risk behaviors appear to be inadequate for producing sustained adherence to cancer prevention recommendations (Lyons et al., 2016). As smartphones become more available, they provide a promising means of implementing and disseminating cancer prevention interventions to a wide range of individuals at a low cost. In the USA, 68.0% of adults owned a smartphone in 2015. Among ethnic groups, AAs, a population that is disproportionately affected by obesity and breast cancer disparities, have the highest smartphone ownership (68.0%) (Pew Research Center, 2015).

There has been a proliferation of lifestyle-oriented mobile apps, however, few have targeted users. Conducted in three phases, the present study was designed to develop an educational intervention to promote adherence to cancer prevention recommendations among AA breast cancer survivors (BCSs) and to evaluate the feasibility and acceptability of delivering the intervention through a smartphone app. The study phases include: 1) a mixed-methods assessment of the lifestyle needs and experiences of the targeted population (2013–2015); 2) a qualitative study, which is the subject of this report, to elicit stakeholder input to guide intervention development (2015–2016); and 3) a feasibility study piloting the app to assess its usability (2018).

## METHODS

The research protocol, *A Community-Engaged Approach to Developing a Mobile Cancer Prevention App (mCPA)*, has been described elsewhere (Smith et al., 2016). Briefly, the study engaged members of a breast cancer support group, Survivors Involving Supporters to Take Action in Advancing Health (SISTAAH) Talk, in a partnership to create content for a smartphone app. The outcome of this study will be a theory-based, culturally tailored, mobile app for testing and future dissemination and implementation.

### Mapping Intervention Development Framework

**Figure 1:**
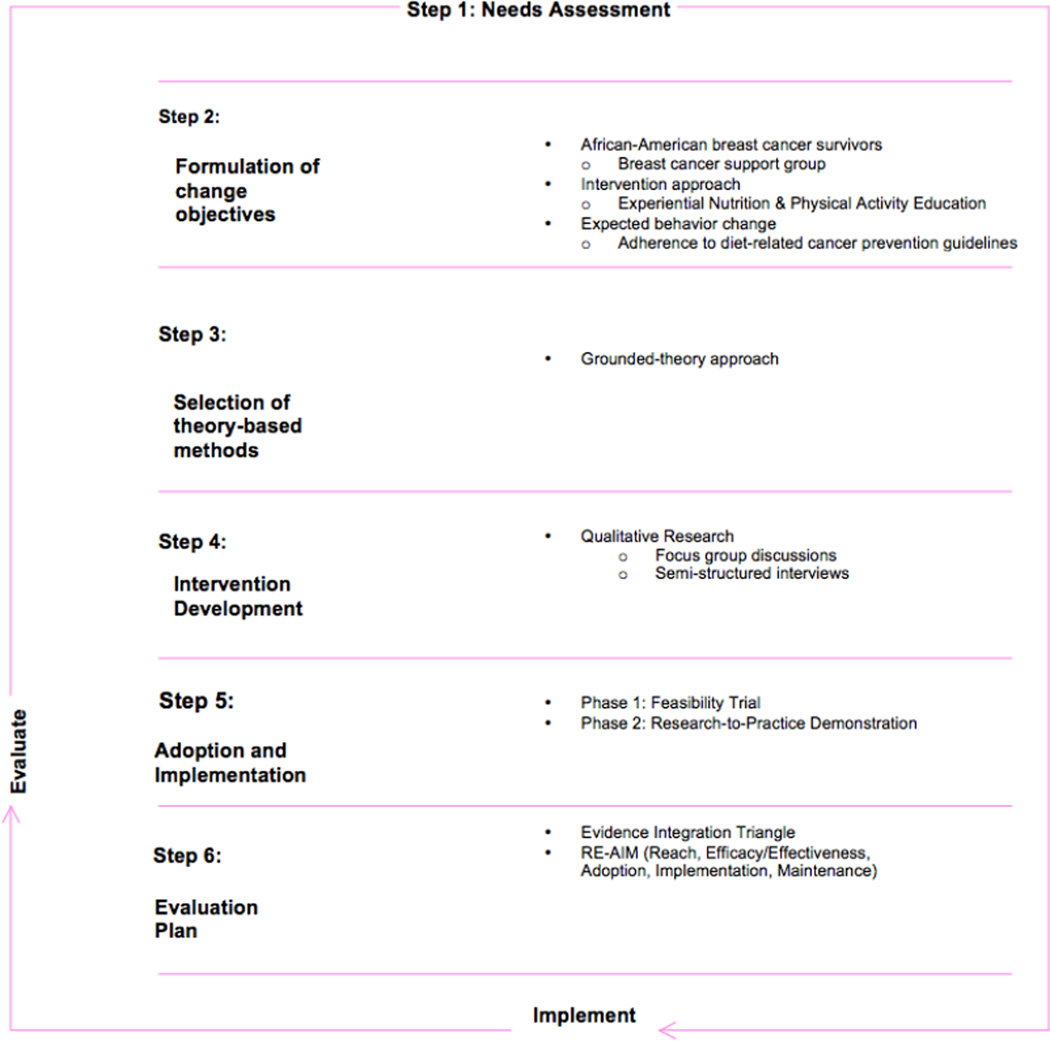
SISTAAH Talk App Intervention Map

Through intervention mapping, investigators partnered with the SISTAAH Talk breast cancer support group to conduct the study. Consistent with community-based participatory research (CBPR) principles (Smith et al., 2015), intervention mapping was selected as a framework to guide development of the intervention (Corbie-Smith et al., 2010). Intervention mapping is an iterative process that begins with a needs assessment and continues by fostering collaborations with stakeholders during intervention development, implementation, and evaluation (McEachan et al., 2008). This systematic process combines theory, empirical scientific literature, and community data to develop culturally appropriate interventions (Bartholomew et al., 1998). The SISTAAH Talk app heuristic intervention map (Figure 1) outlines an iterative process that includes six steps.

Step 1, Needs Assessment, included a literature review, secondary data analysis, lifestyle assessment, and focus group discussions. The *literature review* established that, although lifestyle changes can reduce the risk of recurrence by one half and the risk of breast-cancer associated mortality by one third (Howell et al., 2014), many BCSs do not engage in such strategies. Lifestyle factor data from the Behavioral Risk Factor Surveillance System (BRFSS) revealed three disparity risk categories for AA women: 1) obesity (35.7% vs. 23.7% for whites); 2) inadequate fruit and vegetable consumption (12.6% vs. 17.4% for whites); and 3) physical inactivity (63.8% vs. 50.3% for whites) (Centers for Disease Control and Prevention (CDC), 2015).

In a *secondary data analysis* of the National Health Interview Survey Cancer Control Supplement, the health-related quality of life for female AAs aged 35 and older (n=62) was compared to AA female survivors of other cancers (SOCs) (n=74) and to AA women of the same age with no history of cancer (NHCs) (n=1,566) (Smith et al., 2016; Claridy et al., 2016). There were no statistically significant differences between BCSs and NHCs, but SOCs reported poorer mental health relative to NHCs [3.3 t-points 95% CI 0.6 – 5.9]. A comparison of differences between SOCs and NHCs showed that there were three physical health items in which SOCs were more likely to report poorer physical health relative to NHCs (ability to carry out physical activities [OR=3.4; 95% CI 1.7 – 6.7], level of fatigue [OR=2.0; 95% CI 1.1 – 3.7], and level of pain [OR=3.3; 95% CI 1.3 – 3.9]).

AA BCSs (n=240; mean age: 56.9 years; standard deviation [SD]: 11.8; range: 25–92 years) completed a *lifestyle assessment* survey. More than half were overweight/obese (68.0%); did not limit portion sizes to control weight (89.0%); consumed <5 vegetables and fruits/day (75.0%); and had >5 servings of red (75.0%) and processed meats/week (94.0%).

Participants in four *focus group discussions* (n = 42; mean age: 45.7 years; standard deviation [SD]: 7.9; range: 35–75 years old) identified barriers to and intervention approaches for enhancing dietary intake. Themes emerging from content analysis converged into the following categories: “talk” as central; peer-facilitated sessions; support group approach; no “pamphlet only” control group; “hands on” or interactive nutrition education; supporters (co-survivors); and community-based (not “community placed”) research (Smith et al., 2015).

Step 2, Formulation of Change Objectives was accomplished through contributions from a Community Advisory Board (CAB), with representatives from community coalitions and breast cancer support groups in Miami, Chicago, Houston, Los Angeles, and Philadelphia (Table 1). Employing the iterative characteristic of CBPR, the CAB formulated objectives for lifestyle changes based on results from the previously described needs assessment and AICR prevention guidelines for cancer survivors (WCRF/AICR, 2007). These include: 1) Be as lean as possible without becoming underweight; 2) Be physically active for at least 30 minutes every day to help prevent cancer and prevent recurrence of cancer; 3) Avoid sugary drinks and limit consumption of energy-dense foods (particularly processed foods high in added sugar, low in fiber, or high in fat); 4) Eat more of a variety of vegetables, fruits, whole grains, and legumes such as beans, taking up at least 2/3 of the space on your plate; 5) Limit consumption of red meats (such as beef, pork, and lamb) and avoid processed meats, taking up only 1/3 or less of your plate; 6) If consumed at all, limit alcoholic drinks to two for men and one for women a day; and 7) Limit consumption of salty foods and foods processed with salt (sodium) by substituting herbs and spices high in phytochemicals (e.g., basil, turmeric, paprika, thyme, and dill).

For Step 3, Theory-Based Methods, the health belief model (HBM) and theory of planned behavior (TPB) were selected to undergird the SISTAAH Talk app. The HBM suggests that health-related cognitions for determining behavior considers belief of BCSs that lifestyle behaviors affect breast cancer recurrence, how severe the recurrence would be, and the cost/benefits of lifestyle change (Glanz et al., 2008). The TPB posits that health behavior is affected by past breast cancer experience and social norms (i.e., lifestyle practices) more so than beliefs (i.e., a link between breast cancer recurrence and lifestyle) (Ajzen, 1991). Components of the HBM and TPB that support app features are outlined in Table 2.

Step 4, Intervention Development, the subject of this report, included participatory engagement. SISTAAH Talk members reviewed themes from focus group discussions during three telephone support group meetings with the principal investigator. They then engaged with researchers in videotaped and audiotaped experiential cooking and exercise sessions, which will serve as content for the app.

A feasibility trial and research-to-practice demonstration (Step 5, Adoption and Implementation) and Evaluation (Step 6), assessed through the Reach, Efficacy/Effectiveness, Adoption, Implementation, and Maintenance (RE-AIM) framework (Glasgow et al., 2004), will be completed. Investigators and participants anticipate development of an acceptable, feasible, and accessible mobile app, which will be available for intervention testing in 2018.

### Study Population

Established in 1995 at the University of Miami Sylvester Comprehensive Cancer Center as the first breast cancer support group for women of color in South Florida, SISTAAH Talk has a goal of providing a forum for African-Americans to communicate about and make sense of their diagnosis and treatment in order to achieve improved physical and mental health outcomes. SISTAAH Talk includes women from across Miami-Dade and Broward counties, reaching an average of 20 women each month through education, outreach, and research.

A purposefully selected sample of 12 SISTAAH Talk members, treated for >1 year for Stages I-IIIc breast cancer, 75 years of age or younger, and English speaking/writing, were identified by leaders of the support group as role models or “coaches” to participate in developing mCPA content. Each applicant was interviewed by the principal investigator and support group facilitator to determine their comfort level in participating in focus group discussions, semi-structured interviews, and development of app content (e.g., video taping cooking demonstrations and physical activity).

### Procedures

The Institutional Review Board of Augusta University approved this research plan. Following informed consent, SISTAAH Talk coaches engaged in experiential educational sessions (e.g., cooking and exercise) and audio- and videotaped facilitated discussions. Cooking demonstrations followed cancer prevention guidelines relative to nutrition and dietary intake (e.g., portion control and weight control, vegetables and fruits, red and processed meats, and whole grains). Exercise sessions focused on guidelines specific to physical activity (e.g., 150 minutes per week).

Qualitative methods were used to determine preferences related to cancer prevention. Following the experiential educational sessions, focus group discussions (FGDs) were completed to generate ideas and determine app content. Individual semi-structured interviews (SSIs), which included an open set of questions that allowed new ideas to evolve based on participant responses, were completed to determine barriers to lifestyle modification, potential strategies to address them, and desired app components. The principal investigator, a BCS experienced in qualitative data collection methods, facilitated the FGDs and SSIs. Sample size was determined based on the principle of saturation, which suggests that, with as few as four discussions, no additional information will be obtained (Carlsen and Glenton, 2011). This qualitative sampling technique was used to ensure that perspectives across age groups were obtained.

Six 90-minute FGDs were conducted with the SISTAAH Talk coaches. A semi-structured and open-ended FGD guide was used to ensure adequate content coverage. The interview guide explored: a) perceptions about modifiable risk factors; b) feelings related to inclusion of cancer prevention guidelines in a smartphone app; and c) strategies for lifestyle modification.

Following the three experiential educational sessions, thirty-six 30-minute SSIs were completed with the SISTAAH Talk coaches to assess perceptions about dietary intake and physical activity, to determine strategies for their modification, and to garner users’ preferred components for the app. To learn more about the content of lifestyle apps, SISTAAH Talk coaches reviewed existing smartphone apps. They were then asked during SSIs to evaluate salient features of the apps to determine which, if any, assisted in addressing individual barriers to lifestyle behavior change.

Topic guides for group discussions and individual interviews appear in the Appendix. Prior to their use, the SISTAAH Talk leadership ensured that the FGD and SSI interview guides were written at an appropriate comprehension level. The guides were pilot-tested for appropriateness and language accuracy. Each discussion and interview was digitally recorded, transcribed verbatim, manually coded, and summarized. The FGD and SSI process ended at the point of saturation or when the collection of new qualitative information no longer shed light on the issues under investigation.

### Theoretical Approach and Data Analysis

Data analysis was guided by a grounded-theory approach to allow categories, themes, and patterns to emerge. This tactic, which uses an inductive method to generate substantive ideas that suggest more focused research questions (Corbin and Strauss, 2008), served as the theoretical basis to formative data analysis. Data were analyzed using Qualitative Content Analysis (Schreier, 2012). Coding steps included developing preliminary themes, creating additional codes based on themes that arose, developing non-substantive codes, and producing detailed codes for analysis of specific topics. NVIVO 10 (qualitative data analysis computer software) was used to facilitate the coding process (i.e., assessing the degree of agreement/disagreement across themes and calculating inter-rater reliability scores) (2015). Recurring themes were identified, the research team came to a consensus on coded themes, and themes were summarized for analysis.

## RESULTS

A total of 12 SISTAAH Talk coaches participated in the study (Table 3). Participants were more than 50 years old (60.0%), college educated (75.0%), widowed/divorced (42.0%), and earned an annual income $25,000 or higher (75.0%). The mean period since breast cancer diagnosis was 8.7years.

Data were generated from FGDs and SSIs. Recurring themes that emerged were organized into three categories: 1) perceptions about modifiable risk factors; 2) strategies related to cancer prevention guideline adherence; and 3) app components to address barriers to lifestyle modification.

### Perceptions about modifiable risk factors

In general, FGD participants had a clear concept and understanding of the cancer prevention guidelines specific to dietary intake and physical activity.

### Dietary intake

Racial-ethnic disparities in modifiable breast cancer risk factors (obesity and low consumption of fruits and vegetables) are large and persistent, particularly between White and AA women (CDC, 2015). The comments of one BCS reflects this understanding:

“I have to say that as cancer survivors, we have the choice of making our lives better. Like we have the choice of making decision for ourselves, that’s really a benefit for me. I feel like choosing to eat well; to eat less meat, more vegetables and fruits I think it’s best for us. I enjoy eating well because healthy food tastes really well.”

The prevalence of overweight or obesity among AA women is 82% relative to 63% for white women (NCHS, 2016). Obesity and weight gain after breast cancer diagnosis are associated with poorer outcomes, including decreased quality of life, increased recurrence, breast cancer deaths, and all-cause mortality (McCollough et al., 2011). For overweight and obese women, a sustained weight loss of 10% of initial weight reduces risk of recurrence of a new primary breast cancer. Comments by one participant reflect this perception:

“Well, it’s something that our people have a big problem with. Sometimes when I tell people I am trying to make a change, they say, ‘why you doing that?’ Let’s face it, I am not sure that I can do anything about it, but I think if I do, it could save my life.”

Adherence to diet-related cancer prevention guidelines is relevant to primary prevention and recurrence of breast cancer (ACS, 2016). Limiting consumption of red and processed meats is recommended. Participants shared the following comments:

“I was a little surprised to learn that even with all the talk about how red meat affects cancer cells, that people still eat so much of it.”

“I was saying if I could just try to cut out the red meat because it does not break down in our bodies for two or three days, which makes me constipated, it would help.”

“Processed meats, like sausage, are cheap and you can cook them fast. But, they contain a lot of salt, a lot of fats. When we are buying those things from the store, we have no idea of what’s in them.”

Consuming five or more fruit and vegetable servings daily is recommended for cancer prevention (ACS, 2016). Participants’ perceptions related to this guideline are captured in the following comments:

“One of the things that we can do to eat healthy is eat more vegetables and fruits.”

“I woke up one morning after chemo and was really sick to my stomach. I did a lot of research and decided to gradually start to eat more plant-based foods. Two weeks turned into two months and now, I’ve been doing it for two years. After one month, I could just feel my body losing all this fat, like it was melting.”

Challenges to meeting this recommendation were reflected in these statements:

“I don’t have trouble eating more vegetables. The problem is not adding butter, gravy, processed cheese, and those things that make them taste good.”

“People generally get offended when you say ‘no thank you’ to food. It’s hard to say no to vegetables cooked with added meat.”

Replacing refined grains with whole grains, portion control, avoiding hidden sugar, and the limited sodium content of food are components of the cancer prevention guidelines. Two statements from a participant illustrate this understanding:

“I have to remember to eat smaller meals, more often. That’s what works for me. I need to learn how to use herbs because they definitely make a difference in how food tastes.” “I would say cooking and eating well is pretty simple. You don’t have to have a lot of ingredients to eat well. It’s not about how much you eat, it’s how well it is prepared and how good it is for you.”

Barriers to adherence to diet-related cancer prevention guidelines were related to access to healthy foods, costs, and cultural food preferences.

### Physical Activity

AA BCSs are less likely to report adherence to physical activity guidelines than women of other races (ACS, 2016). Compared to other races/ethnic groups, AA women have the largest potential reductions in breast cancer risk from physical activity (41%) (Friedenreich, 2010), yet they are generally less physically active and more sedentary than women of other racial/ethnic groups. In the present study, the recommendation of 150 minutes of moderate physical activity per week was well received, as reflected in the following:

“Walking not only helps you physically, you are able to release emotionally, you’re able to share and feel alive”

“The first thing people talk about is fatigue and the lack of energy so by walking and partnering up, you can do it. The challenge is to get out and walk and to have a good time.”

“Walking is aerobic; your heart rate goes up.”

The primary barrier to physical activity for FGD participants was limitations due to physical outcomes related to treatment (e.g., lymphedema (swelling in an arm), arthralgia (pain in a joint) and neuropathy (numbness or weakness)). The following statements captured these barriers:

“I tend to baby my surgical side, I’m very cautious with it. Even to the point when I go to the doctor they told me that you don’t do blood pressure from that side, you don’t do anything from that side, nothing over 5 pounds.”

“When you have nothing in the area, the body tends to shift and close up.”

“I gradually starting to stretch and the beginning up exercise, I was like oh my God I can’t wait to get home and try in private”

“Having a tram flap restricted some of my movement. Sometimes I get very frustrated with that and I just don’t want to do. I realized today, just modify it. I did a lot of things the normal way and when I got to the point where I couldn’t do it, I modified it with and it was a revelation to me.”

For BCSs, physical activity improves fitness and alleviates cancer-related fatigue and enhances physical functioning (Knobf et al., 2014). Another theme that arose from the FGDs as a secondary barrier to physical activity was fatigue. According to one BCS:

“Since my chemo ended two years ago, I still have problems with fatigue. When I get home from work, I am really too tired to exercise.”

For some respondents, closely related to fatigue was the extreme hot temperature experienced in South Florida as a barrier to physical activity, as illustrated in one BCS remarked:

“Living in the hottest place in the country [Miami], I hate to sweat. Exercise wears me out.”

### Strategies related to cancer prevention guideline adherence

Participants shared the following strategies for promoting guideline adherence:

“*The feedback from the leader was really encouraging.”*

“Have two pairs of walking shoes and leave one at work so that when a colleague says ‘let’s go walk’ you won’t say that you don’t have any shoes.”

“I wasn’t able to go the complete distance but the distance I went was good for me. I bonded with two of my sistaahs and I enjoyed it. Exercising with another breast cancer survivor really helps.”

“If you have lymphedema, you have to be careful about using heavy weights [for strength training] and you need to wear your sleeves because it’s very important.”

### App components to address barriers to lifestyle modification

Table 4 summarizes the main findings from the SSIs. Strategies were categorized as major themes for inclusion in the mobile cancer prevention app. Components were selected based on apps reviewed by participants. Many participants commented on the need for social support and advice from experts. Further, they wanted the app to be easy to operate, include a tutorial, link to related resources, and operate at no cost to users. Culture and tradition were identified as major themes for lifestyle modification. Related to food, participants wanted the app to include soul food, southern, and Caribbean recipes; for physical activity, they felt that simple, easy-to-do exercises should be the focus.

## DISCUSSION

Twelve AA BCSs (coaches) with a mean age of 50 years, participated in a qualitative study to elicit stakeholder advice to guide the development of a mobile cancer prevention app. Responses from participants were organized into 3 categories: 1) perceptions about modifiable risk factors; 2) strategies related to adherence to cancer prevention guidelines; and 3) app components to address barriers to adherence. Participants were aware of the racial/ethnic disparities that exist in modifiable lifestyle risk for breast cancer, and they understood the importance of choosing to eat healthy, avoiding red and processed meat, consuming more fruits and vegetables, and reducing meal portion sizes. They also understood the importance of walking and aerobic exercise. Barriers to meeting dietary recommendations were making food taste good, access to healthy foods, costs, and preference for cultural foods. For exercise, barriers include limitations from complications of cancer treatment such as lymphedema, joint pain, and neuropathy. The strategies listed for adhering to cancer prevention include partnering with survivors to exercise, having an extra pair of walking shoes for convenience, wearing sleeves, being careful about using heavy weights in cases of lymphedema, and recipes for healthy meals. The app components included educational materials about WCRF/AICR prevention guidelines, a diary and reminders, a BMI calculator, links to social media, internet educational videos, and flags for lapses.

There has been a proliferation of health-related apps, especially ones targeting exercise, diet, and weight. The advantages that web/mobile phone apps hold over standard face-to-face programs include ready availability, less burden, acceptability by target populations, greater program adherence, low cost, self-monitoring, and reaching a large target population. A limitation is determining how to design an appealing app that will maintain user engagement over time (Pelligrini et al., 2015). Health promotion interventions should be culturally appropriate and tailored to meet the needs of the targeted population (Ansa et al., 2015).

The present study is among the first to consider the socioeconomic and cultural needs as well as the health challenges of its intended users in guiding the development of a mobile lifestyle app. This approach is likely to enhance user engagement over time and increase adherence to recommendations for cancer prevention. Previous studies involving technology-based interventions have shown that individually customized messages are more effective than non-customized messages in improving self-efficacy and dietary behaviors (Kroeze et al., 2006). The small sample size and non-generalizability of results to other breast cancer groups are limitations of the study.

## CONCLUSIONS

Among AA BCSs, complex cultural and socioeconomic issues may hinder optimal adherence to cancer prevention guidelines. General prevention measures may not be sufficient for eliminating disparities that exist as a result of such issues. Researchers should work collaboratively with survivors and support groups to ensure that interventions that are developed and tested are relevant and practical in meeting the needs of this group. The findings from this study will assist investigators in the development of a mobile cancer prevention app with features that are usable, acceptable, and accessible to AA BCSs.

## Figures and Tables

**Table 1:** Community advisory board representation

Community Coalitions & Support Groups by City
**Miami**
Florida Resources for Enhancing & Sustaining Health
SISTAAH Talk
**Chicago**
National Black Leadership Initiative on Cancer
Sisters Working it Out
CT Joiner Foundation
**Houston**
National Black Leadership Initiative on Cancer
Angels Surviving Cancer, Inc.
Reconstruction of a Survivor
**Los Angeles**
Black Women for Wellness
Sisters Breast Cancer Survivors Network
Celebrate Life
**Philadelphia**
National Black Leadership Initiative on Cancer
Women of Faith and Hope
Linda Creed Breast Cancer.Org

**Table 2:** SISTAAH Talk app theoretical components

Component	Health Belief Model^a^			Theory of Planned Behavior^b^	
	HBM1	HBM2	HBM3	TPB4	TPB5
Education	✓	✓	✓		
Instructions			✓		
Goal setting					✓
Social support				✓	
Provide feedback					✓
Prompt review					✓
Self-monitoring		✓			✓
Teach use of cues		✓			
Action planning				✓	

a Health Belief Model: HBM1 (perceived costs); HBM2 (health benefits); HBM3 (cues for action).

b Theory of Planned Behavior: TPB4 (subjective norms/social support); TPB5 (behavior control).

**Table 3:** Sociodemographic characteristics of SISTAAH Talk coaches

Characteristics	Participants (n=12)

**Mean age, years (range)**	50 (40–72)

**Educational Level**	
High school or less	3 (25.0)
College	7 (58.3)
Graduate	2 (16.7)

**Marital Status (percent)**	
Single	3(25.0)
Married	4(33.0)
Widow/Divorced	5(42.0)

**Annual Income (%)**	
$0-$24,999	4(33.0)
$25,000-$49,000	5(42.0)
≥$50,000	3(33.0)

**Mean years (range) since diagnosis of breast cancer**	8.7 (2–26)

**Table 4:** Strategies and participant app component preferences

App Component	Strategy	Quotes
WCRF/AICR cancer prevention guidelines	Educational materials	*“Because as a cancer survivor, I think we need* *that kind of education. Because there are so many* *programs teaching how to work out; but as breast* *cancer survivors, need special education that will* *not traumatize our bodies.”*
Food and exercise diary	Dietary intake and physical activity progress	*“I want an app to chart or journal what I have* *been eating and doing—to give me ideas and* *guidance.”*
Email reminders	Feedback Instructions	*“I like email reminders and tips for eating every* *day, reminding me to drink water, to eat fruits* *and vegetables.”*
Body mass index calculator	Self-monitoring of body weight	*“The best app is one that calculates how fat you* *are. Having a BMI calculator is a must!’*
Healthy weight range	Self-monitoring of body weight Goal setting	*“Healthy weight in my community is different* *from for other women. I need to know how much I* *should weight to prevent my breast cancer from* *coming back—I need a goal.”*
Energy/calorie requirement calculator	Self-monitoring of portion control Goal setting	*“Apps that have restaurant information and the* *calculation of the calories for any of the foods* *you eat are best.”*
Food group recommendations	Self-monitoring of fruits and vegetables; red and processed meats; whole grains	“*It would help if the app allowed me to track my* *progress—tell me if I am eating the right number* *of foods to prevent cancer.”*
Recipes—pictures of foods, YouTube videos	Educational materials Instructions	*“The ones (apps) with links to other websites was* *very good.”*
Exercise—instructions, YouTube videos	Educational materials Instructions	*“More information, especially about physical* *activity.”*
Tracking negative thoughts/stress	Stress reduction	*“I learned how to release my stress, to breathe* *that’s a good thing and also not to stress our* *body, to go further than we can. We need to* *include this in the app.”*
Links to FaceBook, Twitter	Social support Feedback	*“Another thing I like is when you can talk with* *certain people—people going through what you* *are going though; experts like nutritionists and* *fitness experts.”*
Internet website links	Resources	*“If it’s too much information, someone like me* *who is new to using apps, if it’s too technical, the* *user will give up.” It’s important to make the app* *‘user-friendly’ and link out to other resources.”*
Reminders to log food and activity	Self-monitoring Goal setting	*“The best app is one that communicates with* *you—that sends you reminders to track your* *progress.”*
Flags for lapses in diet and physical activity goal adherence	Self-monitoring Goal setting	*“Once I set a goal, I usually stick to it. But some* *things that I think that I am doing right, I am not* *really doing right. I need someone to tell me.”*

## APPENDIX

**Table:** Focus Group Discussion Topic Guides

**Physical Activity**
What did you expect to get out of today’s walk (strength training) (yoga) demonstration? Were you looking forward to it? Why or why not? What did you like the most about the walk (strength training) (yoga) demonstration? What did you like least about the walk (strength training) (yoga) demonstration? Do you have any limitations or problems related to walking (strength training) (yoga)? If yes, please describe them. Tell us what you learned about walking (strength training) (yoga); will it motivate you to incorporate it into you daily activities? How did you feel about partnering with a breast cancer survivor for walking (strength training) (yoga)? Were there advantages/disadvantages to partnering? What did you gain from today’s walking (strength training) (yoga) demonstration? Would you advise breast cancer survivors to walk (strength training) (yoga)? Why or why not?
**Dietary Intake**
What did you expect to get out of the cooking (grains) (red meat) (fruits and vegetables) demonstration? What did you like most about the cooking (grains) (red meat) (fruits and vegetables) demonstration? What did you like least about the cooking (grains) (red meat) (fruits and vegetables) demonstration? Tell us how you feel the affects of how you eat and breast cancer? How relevant are the cancer prevention guidelines to the foods that you select? What would you say are the main challenges you have faced in changing your eating habits? Why is reading food labels important to you? What changes to your diet, if any, have you made since your breast cancer diagnosis? How do you feel about letting people around you know that you are trying to change the way that you eat? What are some of the ways that you have been able to get the kind of support that you need to make changes to your diet?

**Table:** Semi-Structured Interview Topic Guides

**Internet Usage**
Do you have daily access to the Internet? If so, do you use it to access information about diet/nutrition? Physical activity? Do you have a smartphone? Do you use apps on your smartphone? If so, do you use apps to obtain information about diet/nutrition? Physical activity? How comfortable are you in using a computer or smart phone to obtain information about diet/nutrition? Physical activity? Which features are important to you to include on the SISTAAH Talk app? How do you feel about developing the SISTAAH Talk app? What do you hope to gain from this experience?
**Smartphone Apps**
What type of smartphone do you have? Android or iPhone? Did you complete the assignment to test health and fitness apps? How many apps did you review? (total number) If yes, did you review diet or nutrition apps? If yes, did you review physical activity or diet apps? What did you like *most* about the apps that you tested? What did you like *least* about the apps that you tested? Of the apps you reviewed, which features would you like to see on the SISTAAH Talk app? If no, please tell me why you did *not* test health and fitness apps? Now, I would like for you to tell me how comfortable you were testing the smartphone apps. On a scaled of 1 to 10, where 1 is not very comfortable and 10 is extremely comfortable, how comfortable were you in using health and fitness apps? Would you say that your comfort level was a 1 _ _ _ _ _ _ _ _ or 10? Based on your response, what do you feel is needed to improve your comfort level? Is there anything else you would like for us to consider in developing the SISTAAH Talk app?
**Experiential Educational Sessions**
Did you find the educational sessions useful in managing your dietary intake? How did you feel about the SISTAAH Talk discussions following the cooking demonstrations? Did participating in developing content for the app inspire you to do more physical activity? Why or why not? If so, did you set a physical activity goal; if yes, what was the goal that you set? Did the content of the cooking demonstrations fit your needs? Please explain how your needs were met. Did the content of the exercise demonstrations fit your needs? Please explain how your needs were met. How confident do you feel about the information that you learned during the sessions? Were the cancer prevention guidelines well explained? If not, which components do you need more information about? After participating in activities to develop the app, are you inspired to eat healthier? To exercise more? Why or why not? Which components inspired you and how were you inspired?
